# The Next Generation Becomes the Now Generation

**DOI:** 10.1371/journal.pgen.1000906

**Published:** 2010-04-08

**Authors:** Diego A. Martinez, Mary Anne Nelson

**Affiliations:** Department of Biology, University of New Mexico, Albuquerque, New Mexico, United States of America; Progentech, United States of America

In recent years, several so-called next-generation DNA sequencing platforms have begun to challenge the well-established Sanger sequencing method. In two important ways—cost and speed—these next-gen technologies provide improvements over Sanger sequencing. Several technical drawbacks (short read length, lack of paired end reads, and quality problems, particularly with homonucleotide stretches [1]), however, render assembly difficult and limit the use of post-Sanger sequencing. These obstacles limited the effective use of next-generation sequencing to the sequencing of prokaryotes [2], the resequencing of individuals [3], and transcriptomics studies, recently termed RNA-Seq [4] and effectively precluded de novo eukaryotic sequencing. Realizing the shortcomings of next-generation technology, manufacturers have continued to improve the read length and have recently implemented paired end methods. Capitalizing on these improvements, the publication by Nowrousian et al. describes the team's success in completely bypassing Sanger sequencing to produce a de novo assembly (to draft quality) of a complete genome, that of the filamentous fungus *Sordaria macrospora* [5], using Solexa sequencing-by-synthesis and 454 pyrosequencing.

The technical merits of this publication make it an excellent starting point for future genome sequencing using post-Sanger platforms. The assembly phase has been a particular sticking point for de novo genome sequencing in eukaryotes, as the complexity of the genomes makes it difficult to correctly place short reads. By sequencing to high depth (nearly 100 times the length of the genome), the authors were able to pull the assembly together in large pieces (contigs) and obtain a reasonable N50 = 117 kb (defined as the smallest length of the longest contigs that cover 50% of the genome). The authors also experimented with different levels of coverage and different combinations of reads to produce assemblies of various qualities. They determined that the depth to which *S. macrospora* was sequenced may not be necessary, and that closing gaps with 454 reads resulted in a large improvement. Interestingly, this is similar to the blend of long- and short-insert libraries that were used for the whole genome shotgun version of the human genome project [6]. By leveraging the short inexpensive Solexa reads for the bulk of the genome, the longer 454 reads can add valuable contig order and orienting information and vastly improve quality while dramatically reducing the associated cost. Nowrousian et al. [5] have provided the assembly statistics for various depths and platforms, paving the way for future studies using high throughput sequencing.

The researchers also showed that post-Sanger sequencing technologies can be used to reliably assemble difficult areas of the genome. One region of the genome, that which controls nonself recognition, could have been a particularly troublesome stumbling block. Anastomosis is a process by which hyphae, the thread-like projections of filamentous fungi, fuse and bring genetically distinct nuclei into contact. Fungi from the same species with different *het* (heterokaryon incompatibilty) loci will fuse, but the resulting heterokaryotic cells are subject to either severely restricted growth or cell death. This process has benefits that the authors describe briefly. Although incompatibility has never been observed in *S. macrospora*, the investigators report that the genome contains apparent heterokaryon incompatibility genes, with the twist that the region is inverted and contains duplications of key genes near the ends of the inversion. Such a duplication might be difficult to resolve with short Solexa data and even the longer 454 reads. However, the authors used polymerase chain reaction (PCR) to amplify across the boundaries of the inverted and duplicated region, and end-sequenced the PCR products to confirm the genome structure predicted by the genome assembler Velvet [7]. Given this demonstrated success in resolving a difficult region containing duplicate genes, researchers and physicians can consider the previously unfeasible next-gen sequencing technologies when deciding whether to sequence an entire genome.

The quality of sequence produced, and ability to compare the Sanger and post-Sanger sequence scores, were additional sticking points to relying completely on the lower cost next-gen technologies. On this front, Nowrousian's team gave us a glimpse of the error rate and how it compares to that of Sanger sequencing by choosing several possible frame shifts in predicted coding regions for resequencing. The outcome of this investigation, although based on a small (21 kb total) sample, shows that the next-gen technologies can achieve error rates similar to those of Sanger sequencing. This leaves no obvious reason to use any Sanger sequencing for future whole genome sequencing projects.

## Beer, Wine, and Advancements in Science and Technology

The selection of organism to sequence in this venture was critical, and a wise choice was made. Fungi, as the authors mention, are not only important to broad areas from ecology and agriculture to medicine and biotechnology, but are also important test platforms due to several characteristics of the genomes inherent to the fungal kingdom. Such traits were important in selecting the yeast *Saccharomyces cerevisiae* as the first sequenced eukaryote, a fungus only distantly related to the filamentous *S. macrospora*. Similar attributes are of value here, chiefly low-repeat content (critical for clean assemblies) and manageable size (*S. macrospora* genome of approximately 40 Mb). The low-repeat content in the genome of *S. macrospora* is possibly due to the effect of repeat-induced point mutation or RIP [8], which has been well documented in the closely related *Neurosopora crassa* [9]. The authors suggest that RIP might have been active at some point in its evolutionary history, but that *S. macrospora* may no longer have an active RIP process. Still, by some mechanism *S. macrospora* is able to keep repeat elements low in copy number. In addition, haploid genomes are much more easily assembled because of a lack of allelic heterozygosity. It remains to be seen how amenable large, diploid genomes will be to assembly using similar technologies.

For one other key reason, *S. macrospora* was an excellent candidate for this next-gen sequencing effort. The close relation to *N. crassa* offers both a good companion for comparative genomics as well as a verification of assembly quality, as large sections of the genomes were known to be similar enough to align extensively [10]. This relationship was also used to pull the assembled fragments together and produce a very clean high-quality assembly with few scaffolds (152 in total).

## Terabyte Is the New Gigabyte

**Figure 1:**
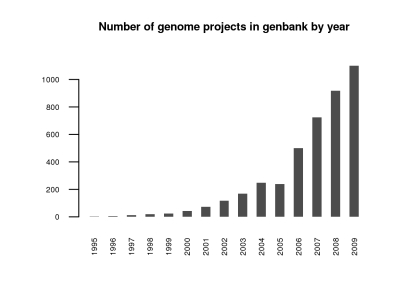
Number of genomes entered into GenBank by year as of September 2009. Adapted from http://www.genomesonline.org/ [11].

**Figure 2:**
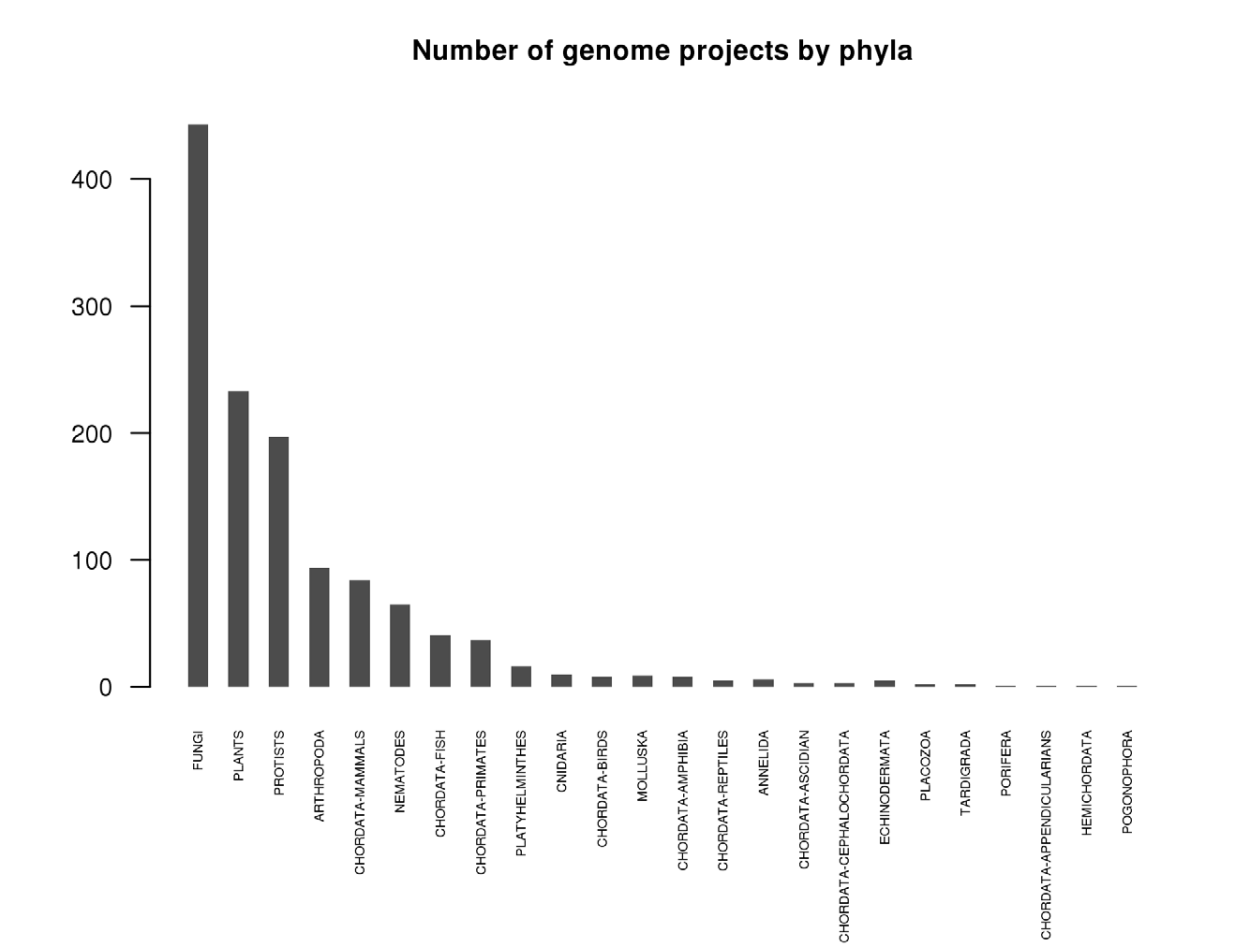
Number of projects per phylogenetic group as of September 2009. Adapted from http://www.genomesonline.org/ [11].

Now that any academic department or perhaps even lab around the world can sequence a draft quality genome inexpensively, the amount of sequence data will predictably explode. While the number of genomes sequenced to date is more than one thousand (Figures 1 and 2) [11]—if we count both eukaryotic and prokaryotic projects—this advancement opens the door to an exponential expansion in the number of available genomes. Can we handle it? The National Center for Biotechnology Information (NCBI) currently deals well with several strains of the same species, but are we ready for individuals of the same strain? While technical hurdles to individual sequencing (the need for multiple copies of the same genome to fragment) remain for single-celled organisms, for fungi, and other eukaryotes with small genomes, this is a likely next level of study. Clearly the expected flood of data and the potential for finding answers to biological questions on this new level make it imperative to develop robust tools for referencing and storing sequence information on an individual by individual basis, and perhaps doing away with the current system of using a single reference genome.

At least for the fungal research community, the quality, cost, and speed of next-gen sequencing technologies are now such that we can sequence at will and add to the rapidly growing list of available fungal genomes, as shown in Figure 2. This may be the case for mammalian genomes as well, as suggested in a recent publication (the giant panda [12]). Still, we have not yet attained the “1,000-dollar genome” widely thought to be necessary for broad medical use in diagnosis and selection of treatments [13].

What is the new next-gen sequencing? One answer to this question might come from Pacific Biosciences Corporation. In a recent publication [14], it appears they are able to detect the addition of a nucleotide to a growing strand of DNA by the polymerase enzyme. This “real-time” sequencing technology may be the next point in the race for fast and inexpensive whole-genome sequencing. Additional companies such as Complete Genomics and Ion Torrent Systems are unveiling new instruments and techniques and it is likely the speed with which data are produced will continue to increase while the costs will decrease. Until then, we will have plenty of data to sift through while we wait.
